# Photosynthetic characteristics, yield and quality of sunflower response to deficit irrigation in a cold and arid environment

**DOI:** 10.3389/fpls.2023.1280347

**Published:** 2023-11-17

**Authors:** Xietian Chen, Hengjia Zhang, Anguo Teng, Changlong Zhang, Lian Lei, Yuchun Ba, Zeyi Wang

**Affiliations:** ^1^ College of Agronomy and Agricultural Engineering, Liaocheng University, Liaocheng, China; ^2^ Yimin Irrigation Experimental Station, Hongshui River Management Office, Zhangye, China; ^3^ College of Water Conservancy and Hydropower Engineering, Gansu Agricultural University, Lanzhou, China

**Keywords:** water deficit, photosynthetic characteristics, yield, quality, comprehensive evaluation, sunflower

## Abstract

In arid regions, deficit irrigation stands as an efficacious strategy for augmenting agricultural water conservation and fostering sustainable crop production. The Hexi Oasis, an irrigation zone situated in Northwest China, serves as a pivotal area to produce grain and cash crops. Nonetheless, due to the predominant conditions of low rainfall and high evaporation, the scarcity of irrigation water has emerged as a critical constraint affecting crop growth and yield in the area. In order to evaluate the effects of deficit irrigation on photosynthetic characteristics, yield, quality, and water use efficiency of sunflower, a two-year field experiment with under-mulched drip irrigation was conducted in the cold and arid environment of the Hexi Oasis region. Water deficits were implemented at sunflower seedling and maturity and consisted of three deficit levels: mild deficit (65–75% field capacity, FC), moderate deficit (55–65% FC), and severe deficit (45–55% FC). A total of six combined water deficit treatments were applied, using full irrigation (75–85% FC) throughout the entire crop-growing season as the control (CK). The results illustrated that water deficit engendered a decrease in leaf net photosynthetic rate, transpiration rate, and stomatal conductance of sunflower compared to CK, with the decrease becoming significant with the water deficit increasing. A mild water deficit, both at the seedling and maturity phases, precipitated a significant enhancement (*p<* 0.05) in leaf water use efficiency. Under mild water deficit, stomatal limitation emerged as the predominant factor inducing a reduction in the photosynthetic capacity of sunflower leaves, while as the water deficit escalated, non-stomatal limitation progressively assumed dominance. Moreover, a mild/moderate water deficit at seedling and a mild water deficit at maturity (WD1 and WD3) significantly improved sunflower seed quality under consistent yield conditions and significantly increased irrigation water use efficiency, with an average increase of 15.3% and 18.5% over the two years, respectively. Evaluations utilizing principal component analysis and membership function methods revealed that WD1 attained the highest comprehensive score. Consequently, a mild water deficit at both seedling and maturity (WD1) is advocated as the optimal deficit irrigation strategy for sunflower production within the cold and arid environment of Northwest China.

## 1 Introduction

Sunflower (*Helianthus annuus L.*), a pivotal global oil crop, ranks fourth in production scale, trailing only to palm oil, soya bean oil and rapeseed oil (Rauf et al., 2017; Mahmood et al., 2019). Due to its notable tolerance to low temperature, salinity and drought, sunflower is well adapted to various environments (Garcia-Lopez et al., 2016; Ramu et al., 2016). In China, as a significant cash crop, sunflower has not only been expansively cultivated but also has critically fostered regional agricultural economic development (Wang et al., 2021). The Hexi Oasis irrigation area, situated within the inland arid zone of Northwest China, is one of the major producing areas of sunflower (Wang et al., 2020). This area exhibits a cold climate, ample sunlight, and pronounced diurnal temperature variations, which are highly conducive to the formation of photosynthetic products and the accumulation of nutrients for the crop. Nonetheless, frequent crop yield reductions occur due to the region’s scarce, unevenly distributed annual precipitation and pronounced evaporation (Wang et al., 2023). Over the past two decades, the Hexi Oasis has averaged a total of 6,628 million m³ in water resources, with per capita water resources approximating 1,270 m³. This figure starkly contrasts with China’s average of 2,100 m³ and the international warning line of 1,700 m³ per capita (Zhao et al., 2023). Moreover, flood irrigation still predominates in most Hexi Oasis sunflower production, leading to an inefficient utilization of constrained water resources. Consequently, developing efficient water-saving irrigation strategies remains imperative for ensuring sustainable sunflower production and mitigating agricultural water stress in the region. (Zhang et al., 2021b; Bai et al., 2022; Zhou et al., 2022).

Deficit irrigation is an efficient water-saving irrigation technique whose core concept is to influence the redistribution of crop photosynthetic products to different tissues and organs by artificially imposing a certain water deficit at a given crop growth stage (El-Bially et al., 2018). This technique aims to minimize the growth redundancy in nutrient organs while maintaining or enhancing economic yield, thereby achieving agricultural water conservation, yield stability, quality improvement and sustainable development (Liu et al., 2016; Chand et al., 2021; Dingre and Gorantiwar, 2021). The key to implementing water deficit irrigation is to quantify the response of crop growth to water stress (Salem et al., 2022; Ramadan et al., 2023). Sunflower is very well adapted to arid and semi-arid environments compared to other crops due to its ability to uptake water from deep soil layers under water stress through a well-developed root system (Connor et al., 1985; El-Bially et al., 2022a). However, pronounced water deficit can markedly inhibit sunflower’s vegetative growth, particularly in the aboveground parts. In turn, a reduction in aboveground biomass can lead to insufficient photosynthetic products, thereby limiting the reproductive growth and ultimately resulting in yield loss (Saudy et al., 2023). Additionally, crops exhibit varying sensitivities to water stress across different growth stages (Jensen et al., 2010). Several studies have indicated that sunflower is particularly sensitive to water stress from early flowering to maturity, and that deficit irrigation during this period can lead to a significant reduction in sunflower yield (Shafi et al., 2013; Garcia-Lopez et al., 2014). Karam et al. (2007) revealed that while water deficit during flowering reduces sunflower yield, it can increase yield during initial seed formation. Sadras et al. (1993) found that water deficit during the early reproductive stages increased sunflower head assimilation and compensated for the decrease in seed number per head by increasing seed size, thereby stabilizing yield. Xu et al. (2017) observed that appropriate reduction in irrigation preceding sunflower sowing favored the biomass accumulation in the later stages, whereas water deficit treatment between budding and flowering stages significantly reduced economic yield. Moreover, several studies reported that deficit irrigation with a 20–40% reduction from adequate irrigation had no significant effect on sunflower yield (Demir et al., 2006; Garcia-Lopez et al., 2016). Therefore, understanding the impact of the degree, period, and duration of water deficit on sunflower growth and yield via multi-year field experiments is pivotal to devise deficit irrigation strategies tailored for efficient sunflower production in a specific region. Photosynthesis is the most fundamental life activity for plant growth, and it is essential for organic matter accumulation and yield formation (Oikawa and Ainsworth, 2016). The exploration of photosynthesis and its influencing mechanisms under abiotic stress conditions persists as a focal point within plant physiology and ecology (Gao et al., 2018). Previous studies have found that water stress induces stomatal closure, diminishing photosynthetic rates in crop leaves, and consequently inhibiting the biomass accumulation (Makhlouf et al., 2022; Shaaban et al., 2023). The reasons for reduced photosynthetic capacity of plant leaves caused by water deficit are usually attributed to both stomatal and non-stomatal limitations (Wang et al., 2018; Zhang et al., 2018). Farquhar and Sharkey (1982) stated that under water stress conditions, the initial response of plant leaves involves stomatal closure. The subsequent decline in stomatal conductance fails to sustain the intercellular CO_2_ concentration (Ci) required for photosynthesis, termed as stomatal limitation of photosynthesis. Conversely, water deficit impairs the photosynthetic activity of plant mesophyll cells (such as reducing the activity of Rubisco and RuBP enzymes) and compromises the structural and functional integrity of photosynthetic organs, inducing a decline in the rate of photosynthesis, referred to as non-stomatal limitation of photosynthesis. The determination of which factor is primarily responsible for the decline in photosynthetic capacity of plant leaves is made by the direction of change in Ci and stomatal limiting values (Ls). When Ci decreases and Ls increases, it is stomatal limitation, whereas when Ci increases and Ls decreases, it is non-stomatal limitation (Cao et al., 2009). Accurately identifying the limiting factors of water deficit on photosynthesis can facilitate comprehension the adaptability of plants to adverse conditions and thus make appropriate irrigation decisions. Generally, under mild and moderate water stress, stomatal limitation has a major influence on photosynthesis, whereas under severe water stress, non-stomatal limitation has a dominant impact on photosynthesis (Flexas et al., 2004; Grassi and Magnani, 2005). Recent studies increasingly spotlight stomatal and non-stomatal limitations of photosynthesis in plant leaves under stress conditions (Campos et al., 2014; Anev et al., 2016). While Zeng et al. (2014) and Ma et al. (2022) explored the photosynthetic limiting factors of sunflower leaves under salinity stress in China’s Hetao Irrigation District, the impact of water stress on the photosynthetic limiting factors of sunflower leaves has garnered minimal attention.

Numerous studies have addressed the response of sunflower yield and water use efficiency (WUE) to deficit irrigation (Karam et al., 2007; Mahmood et al., 2019; Zhang et al., 2021a); however, a limited focus has been cast on the comprehensive effects of deficit irrigation on sunflower photosynthetic characteristics, yield, seed quality and WUE. Notably, the results of previous studies can hardly provide sufficient support for the development of optimal deficit irrigation strategies for sunflower in the Hexi Oasis region due to the different climatic conditions, soil textures and experimental varieties. Moreover, under-mulched drip irrigation, recognized for its thermal insulation, moisture conservation, and WUE enhancement, finds prevalent usage in arid regions; and its combination with deficit irrigation could assure regular crop production whilst maximizing water conservation (Bai et al., 2022; Wang et al., 2023). However, little research has investigated the application of under-mulched drip irrigation combined with deficit irrigation for sunflower production. Consequently, a two-year field experiment of deficit drip irrigation beneath plastic film mulching was conducted in the cold and arid environment of the Hexi Oasis region, Northwest China. This study hypothesized that appropriate deficit irrigation at the seedling and maturity stages of sunflower could improve water productivity and grain quality while avoiding excessive accumulation of vegetative organ biomass. The specific objectives of this study were to determine: (1) the response of leaf photosynthetic characteristics of sunflower to water deficit and its limiting factors; (2) the effect of water deficit on sunflower yield components, quality and WUE; and (3) the optimal water deficit irrigation strategy based on principal component analysis (PCA) and membership function (MF) methods. The results of this study will provide theoretical guidance for the efficient production and scientific irrigation of sunflower in the cold and arid environment of Northwest China.

## 2 Materials and methods

### 2.1 Experimental site description

**Figure 1:**
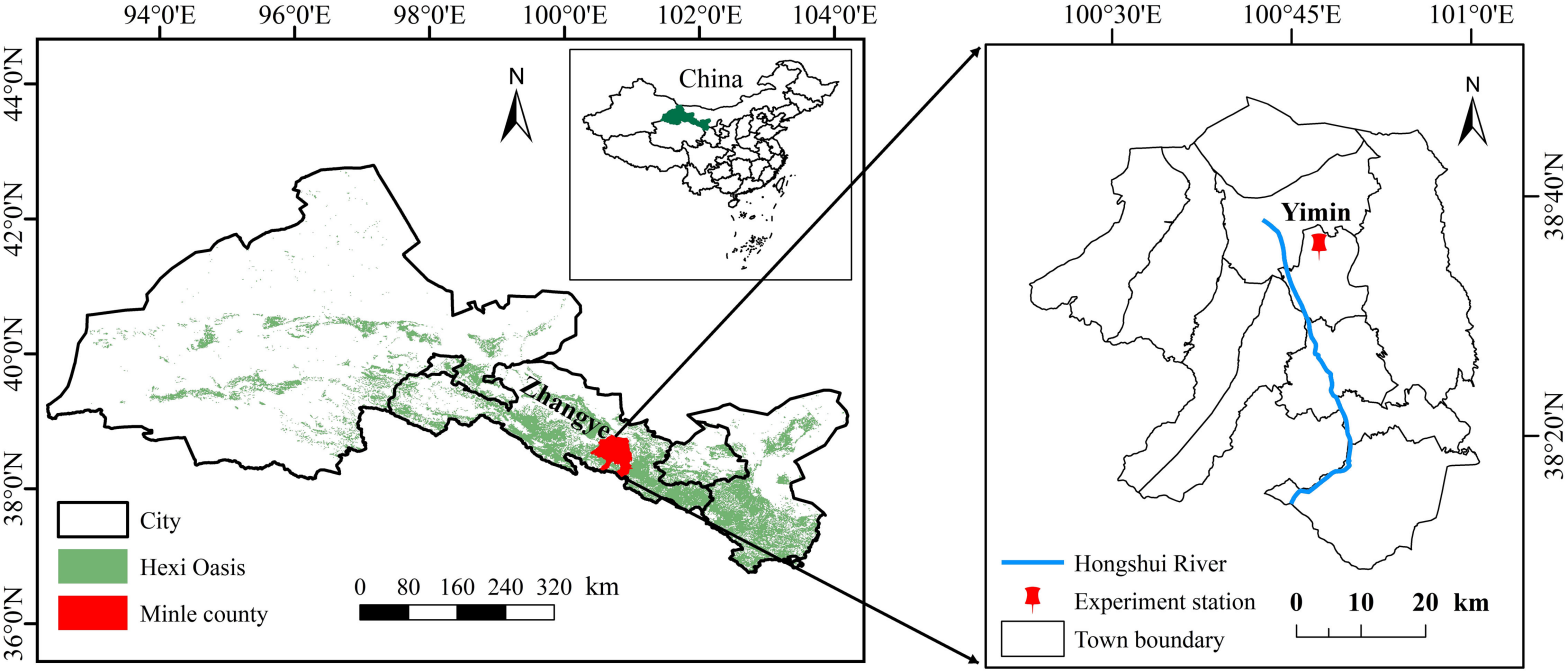
Location of the experimental site.

A field experiment for two sunflower growing seasons (2019 and 2020) was conducted at Yimin Irrigation Experimental Station (100°47′ E, 38°35′ N) in Minle County, Gansu Province of Northwestern China (**Figure 1**). The station is situated in the northwestern inland river basin with a cold and arid climate, which is a typical oasis agricultural irrigation area. The region experiences high evaporation and frequent droughts, with an average annual evapotranspiration of more than 2000 mm, an average annual rainfall of about 200 mm, an average annual sunshine duration of 3000 hours, and an average annual temperature of 6.0°C. The experimental station boasts a soil texture classified as light loam, displaying an average bulk density of 1.41 g·cm^−3^ within the 0 to 60 cm soil layer, a field capacity (FC) of 24% (mass water content), and a pH of 8.5. The contents of soil organic matter, available phosphorus, available potassium, and alkali hydrolyzed nitrogen in the 0 to 20 cm soil layer were 12.8 mg·kg^−1^,13.1 mg·kg^−1^,192.7 mg·kg^−1^, and 63.5 mg·kg^−1^, respectively.

### 2.2 Experimental design

**Figure 2:**
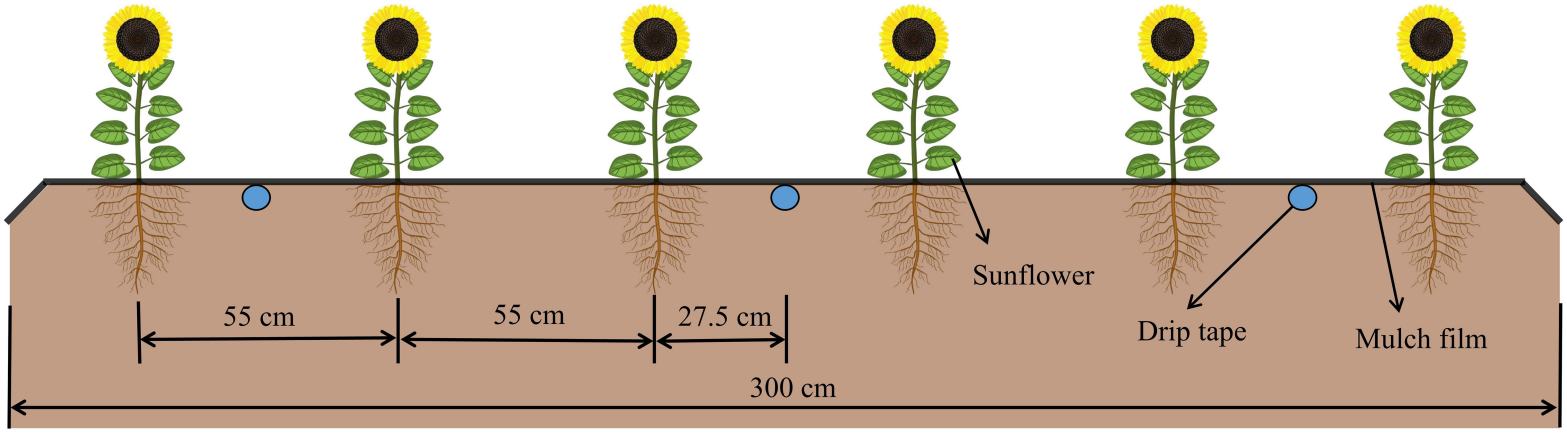
Layout of drip irrigation under plastic film for sunflower.

The experimental crop variety was edible sunflower “JK601” (a primary local variety), sown on 15 April 2019 and 29 April 2020, and harvested on 7 September and 16 September of the corresponding year, with a growing season of 146 and 139 days, respectively. In this experiment, full film coverage was adopted, and the film thickness was 0.01 mm. Sunflowers were manually sown at a depth of 5 cm, with a row spacing of 55 cm and a plant spacing of 40 cm. Drip irrigation under plastic film (**Figure 2**) was utilized as the irrigation method, wherein the drippers were placed 30 cm apart, and a designed flow rate and rated working pressure were maintained at 2.5 L/h and 0.1 MPa, respectively. The irrigation timing and volume for each plot were regulated by the gate valve of the branch pipe and the water meter (accuracy: 0.0001 m^3^). Each experimental plot spanned an area of 24 m^2^ (3×8 m), and a 0.8 m wide separation zone was established between adjacent plots. Additionally, a plastic partition (60 cm deep) was embedded to inhibit lateral moisture movement. Moreover, urea (225 kg·hm^−2^), potassium (300 kg·hm^−2^) and diamine phosphate (300 kg·hm^−2^) were applied as base fertilizer prior to sowing, adhering to the local farmers’ sunflower cultivation standards.

**Figure 3:**
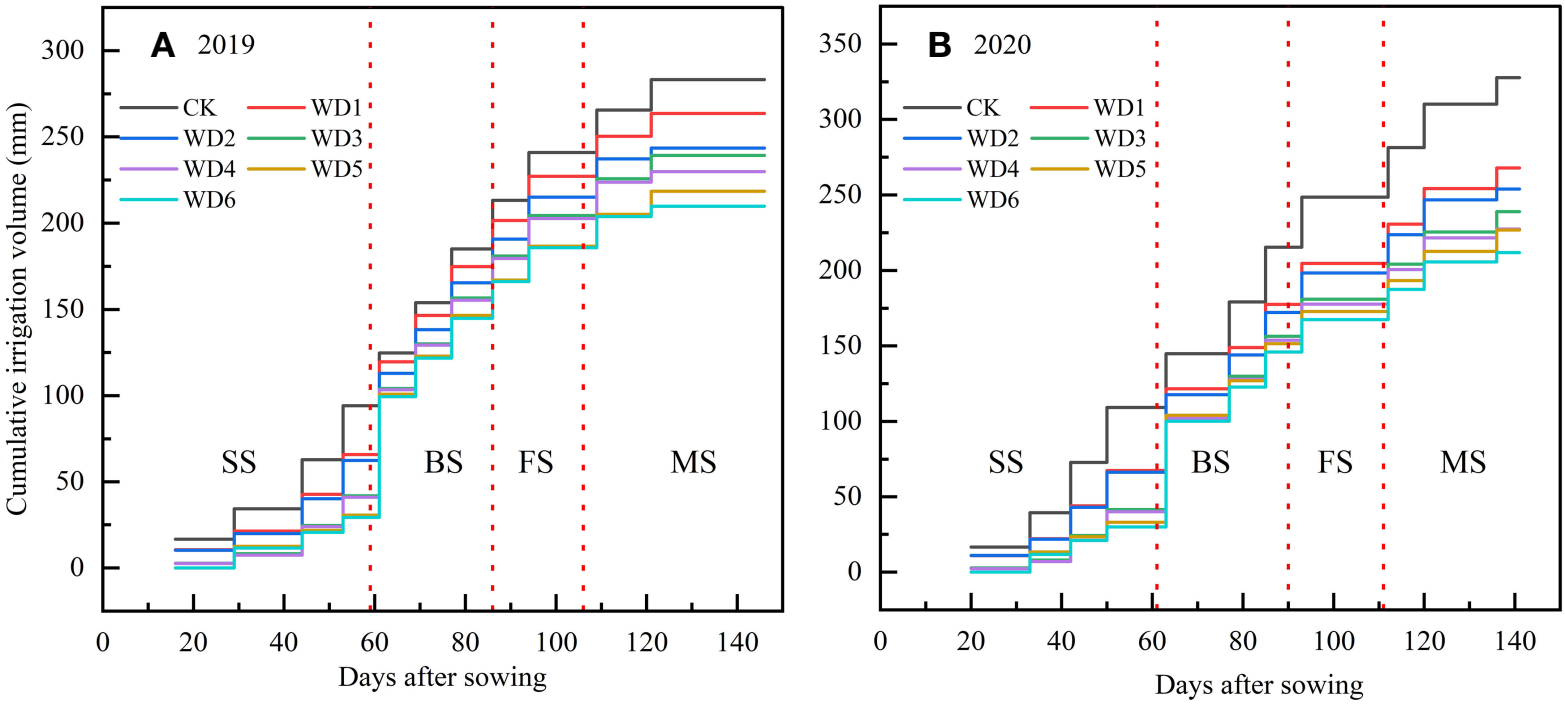
Irrigation time and cumulative irrigation volume of sunflower under different water deficit treatments in 2019 **(A)** and 2020 **(B)**. SS, seedling; BS, budding; FS, flowering; MS, maturity.

According to crop growth characteristics, the entire growing period of sunflower was divided into four distinct phases: seedling (approximately 45 d), budding (approximately 25 d), flowering (approximately 22 d) and maturity (approximately 35 d). Four irrigation levels were established in this experiment, namely full irrigation (75%–85% FC), mild (65%–75% FC), moderate (55%–65% FC), and severe (45%–55% FC) water deficit. Deficit irrigation was designed at seedling and maturity, with mild, moderate and severe deficit irrigation applied at seedling, and mild and moderate deficit irrigation applied at maturity, thereby constituting a total of six water deficit irrigation treatments. Additionally, a full irrigation treatment during the entire growth period was established as a control (CK). The experiment adhered to a randomized complete block design, encompassing three replications and thereby constituting a total of 21 plots. During the water sensitive periods, the sunflower was fully irrigated at both budding and flowering. The specific experimental scheme and the actual cumulative irrigation volumes are shown in **Table 1**; **Figure 3**, respectively.

**Table 1:** Experimental scheme.

Treatments	Design range of soil moisture in different growth stages (% FC)			
	Seedling	Budding	Flowering	Maturity
WD1	65–75	75–85	75–85	65–75
WD2	65–75	75–85	75–85	55–65
WD3	55–65	75–85	75–85	65–75
WD4	55–65	75–85	75–85	55–65
WD5	45–55	75–85	75–85	65–75
WD6	45–55	75–85	75–85	55–65
CK	75–85	75–85	75–85	75–85

WD1, mild water deficit at seedling and maturity; WD2, mild water deficit at seedling and moderate water deficit at maturity; WD3, moderate water deficit at seedling and mild water deficit at maturity; WD4, moderate water deficit at seedling and maturity; WD5, severe water deficit at seedling and mild water deficit at maturity; WD6, severe water deficit at seedling and moderate water deficit at maturity; CK, full irrigation during the whole growth period.

### 2.3 Measurements and calculations

#### 2.3.1 Meteorological data

**Figure 4:**
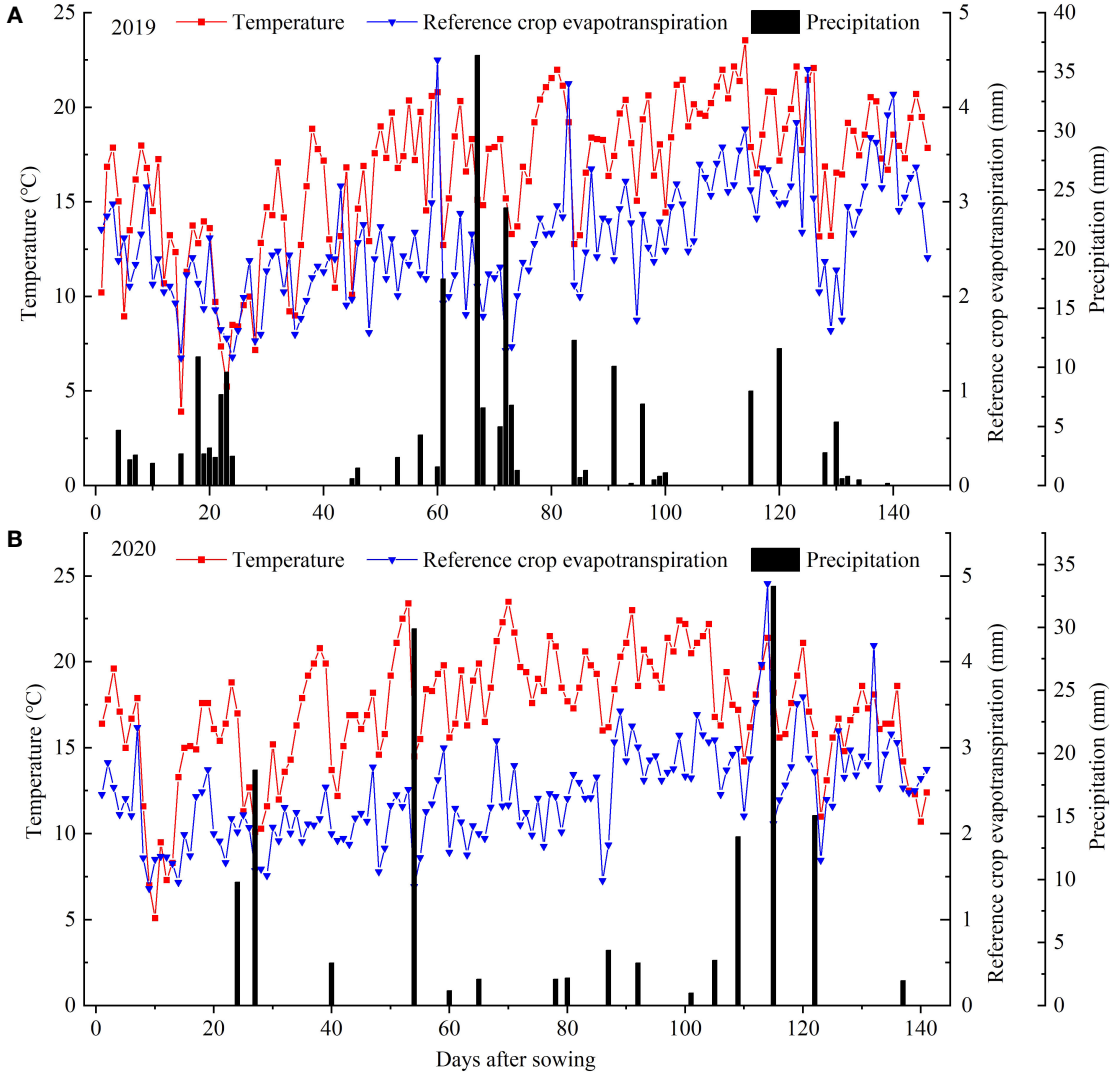
Temperature, precipitation and reference crop evapotranspiration (ET_0_) during the whole growth period of sunflower at the experimental site in 2019 **(A)** and 2020 **(B)**.

Meteorological data for the sunflower growing season were automatically collected by a weather station, located 30 m from the experimental field, encompassing parameters such as precipitation, air pressure, wind speed, air temperature and relative humidity. Daily reference crop evapotranspiration (ET_0_) was calculated using the FAO Penman-Monteith formula (Allen, 1998). **Figure 4** illustrates the variation patterns of daily temperature, precipitation and daily ET_0_ during the sunflower growing season. In 2019 and 2020, the precipitation of the sunflower growing season was 221.9 mm and 147.4 mm, while the effective precipitation (≥5mm) was 166.1 mm and 106.9 mm, respectively. The average daily temperature was 16.6°C and 17.1°C, whereas the average daily ET_0_ was 2.6 mm and 2.4 mm for the years 2019 and 2020, respectively.

#### 2.3.2 Photosynthetic indicators

Photosynthetic indicators were measured *in vivo* using a portable photosynthetic meter (LI-6400XT, LI-COR, Lincoln, NE, USA) 3–7 days subsequent to the deficit irrigation, under clear and cloudless meteorological conditions. Measurements were conducted between 8:00 and 18:00, with a 2-hour interval. Healthy leaves from the same parts of representative sunflower plants were selected for measurement and three leaves were randomly measured in each plot. The indicators measured included leaf net photosynthetic rate (Pn), transpiration rate (Tr), stomatal conductance (Gs), intercellular CO_2_ concentration (Ci) and Ci/Ca (where Ca represents atmospheric CO_2_ concentration). Stomatal limitation value (Ls) is commonly used to determine the limiting factor of photosynthesis and is calculated using the following formula (Berry and Downton, 1982):


(1) Ls=1-Ci/Ca

#### 2.3.3 Yield and quality

After the sunflower seeds had matured, five plants were randomly selected from each plot. A steel tape measure (accuracy: 0.1cm) was used to determine the head diameter (HD), followed by threshing and air-drying of the seeds, weighing the 100-seed weight (HW) and the grain weight per head, and converting to yield per hectare. The crude protein (CP) content of sunflower seeds was determined by the standard Kjeldahl method (Liu et al., 2021b). The crude fat (CF) content was determined by the Soxhlet extraction method (Lei et al., 2020).

#### 2.3.4 Irrigation water use efficiency and leaf water use efficiency

WUE, an important indicator of plant resistance to water stress, has been defined differently at different scales (Cao et al., 2009). In this study, irrigation water use efficiency (IWUE) and leaf water use efficiency (LWUE) were selected to measure the adaptability of sunflower to different water deficits. IWUE and LWUE are defined as follows (Zhang et al., 2018):


(2) $$\text{IWUE}=\frac{\text{Y}}{\text{I}}$$

(3) $$\text{LWUE}=\frac{\text{Pn}}{\text{Tr}}$$

where Y is the sunflower yield (kg·hm^-2^), I is the irrigation volume (mm), Pn is the net photosynthetic rate (μmol·m^-2^·s^-1^), and Tr is the transpiration rate (mmol·m^-2^·s^-1^).

#### 2.3.5 Comprehensive evaluation

In order to determine the optimal deficit irrigation strategy for sunflower cultivation, this study established a comprehensive evaluation system using PCA and MF methods. PCA served to diminish the dimensionality of the evaluation indicators and to derive a new set of independent comprehensive indicators. The MF method, constituting the core of the evaluation system, hinges on the principles of fuzzy mathematics and employs the membership function for comprehensive evaluation. The specific steps to establish the evaluation system are detailed as follows as follows (Chen et al., 2021).

Extract the principal component and calculate the score:


(4) $$F_{i}=U_{1\text{i}}X_{1}+U_{2\text{i}}X_{2}+\cdots +U_{P\text{i}}X_{p}$$

where *F_i_* is the *i*th principal component score, which is also serves as a new independent comprehensive indicator. *p* is the number of evaluation indicators, *X_p_* is the standardized evaluation indicator, and *U_pi_* is the score coefficient.

(2) Calculate membership function values.


(5) $$u(x_{i})=\frac{x_{i}-x_{\min}}{x_{\max}-x_{\min}}$$

where u(*x_i_*) is the membership function value of the *i*th indicator, *x_i_* is the measured value of the *i*th indicator, and *x_max_* and *x_min_* are the maximum and minimum values of the *i*th indicator, respectively.

(3) Determine the weights.


(6) $${\text{w}}_{\text{i}}=\frac{P_{\text{i}}}{\sum_{\text{i}=1}^{\text{n}}P_{\text{i}}}$$

where *w_i_* denotes the weight and *P_i_* denotes the contribution rate of the *i*th comprehensive indicator.

(4) Calculate the comprehensive evaluation values (D).


(7) $$D=\sum_{\text{i}=1}^{\text{n}}\left[u(x_{\text{i}})\times {\text{w}}_{\text{i}}\right]$$

#### 2.3.6 Statistical analyses

Experimental data were processed and calculated using Microsoft Excel 2019 (Microsoft Corp., USA). One-way analysis of variance (ANOVA) and principal component analysis (PCA) were performed using SPSS 20.0 (IBM Corp., USA). Differences in means were compared using the Least Significant Difference (LSD) method at a significance level of p< 0.05. Figures were generated using Origin 2021b (Origin Lab Corp, USA).

## 3 Results

### 3.1 Photosynthetic characteristics

#### 3.1.1 Leaf net photosynthetic rate

**Figure 5:**
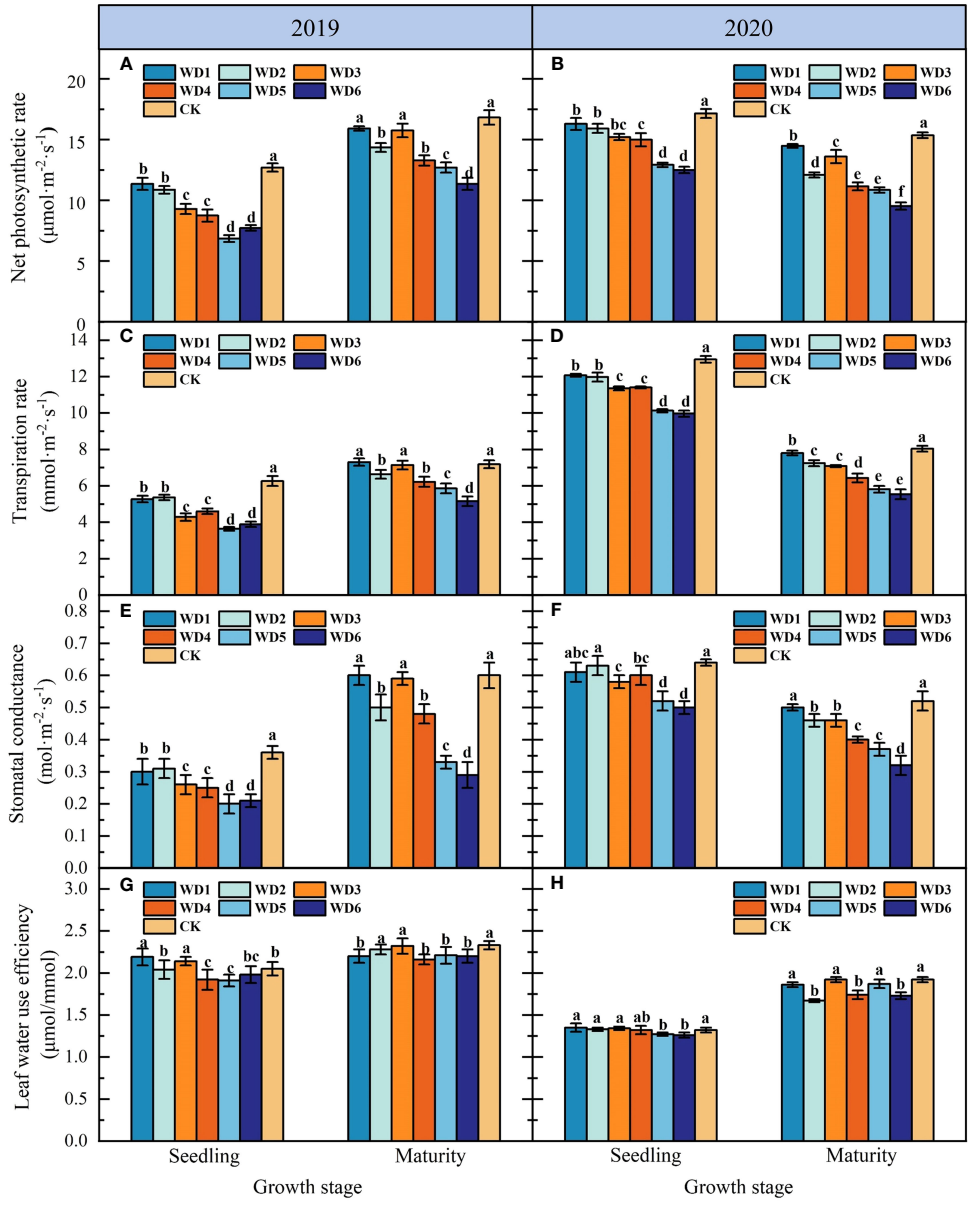
Effect of water deficit on net photosynthetic rate **(A, B)**, transpiration rate **(C, D)**, stomatal conductance **(E, F)** and leaf water use efficiency **(G, H)** in sunflower in 2019 and 2020. Different lowercase letters indicate significant differences at *p* < 0.05. Bars indicate the standard deviations.

**Figures 5A, B** shows the effects of each deficit irrigation treatment on sunflower leaf Pn in 2019 and 2020. As can be seen, deficit irrigation during seedling and maturity in 2019 and 2020 resulted in the decrease in Pn, with a more pronounced decrease concomitant with escalating water deficit. At seedling, Pn under deficit irrigation treatments was significantly reduced (*p*< 0.05) by 4.90%–27.06% and 9.92%–45.98% compared to CK in 2019 and 2020, respectively, with WD5 and WD6 exhibiting the most substantial decline. At maturity, Pn of WD1 and WD3 decreased compared to CK in 2019, but the difference was not significant (*p* > 0.05). Pn of WD2, WD4, WD5 and WD6 were significantly reduced in 2019 and 2020 compared to WD1, WD3 and CK, especially WD6 was reduced by 32.07% (2019) and 37.92% (2020) compared to CK.

#### 3.1.2 Leaf transpiration rate

In the seedling, Tr decreased significantly (*p*< 0.05) in water deficit treatments compared to CK, with the decrease increasing concomitant with the exacerbation of water deficit (**Figures 5C, D**). Specifically, in 2019, WD5 had the lowest Tr, demonstrating a significant decrease of 38.20% compared to CK. Conversely, in 2020, WD6 registered the lowest Tr, with a significant decrease of 31.13% relative to CK. No significant difference (*p* > 0.05) was identified in Tr between the identical water deficit treatments at seedling. At maturity, excluding WD1 and WD3, Tr in the other water deficit treatments decreased significantly by 7.64%–28.45% compared to CK in 2019, while in 2020, Tr across all water deficit treatments decreased significantly within a range of 2.8% to 31.03% compared to CK. Furthermore, Tr in WD2, WD4 and WD6 at maturity was significantly lower than that of WD1, WD3 and WD5 in 2019 and 2020, respectively, suggesting that a moderate water deficit at maturity could significantly reduce leaf transpiration in sunflower.

#### 3.1.3 Leaf stomatal conductance

As shown in **Figures 5E, F**, there was no significant difference (*p* > 0.05) in leaf Gs of sunflower among identical water deficit treatments at seedling. In 2019, Gs in water deficit treatments at seedling were significantly reduced (*p*< 0.05) by 11.40%–47.37% compared to CK. Whereas in 2020, although the Gs of WD1 and WD2 exhibited a reduction relative CK, this difference did not attain statistical significance. Conversely, Gs in the remaining water deficit treatments were significantly lower by 9.38%–23.44% compared to that of CK. Additionally, there was no significant difference in Gs between WD1 and WD3 at maturity compared to CK, while Gs in other water deficit treatments decreased significantly compared to CK. Notably, among them, WD6 had the lowest Gs, which was 51.23% and 36.53% lower than that of CK in 2019 and 2020, respectively.

#### 3.1.4 Leaf water use efficiency

**Figures 5G, H** illustrates the effects of each deficit irrigation treatment on LWUE of sunflower. In 2019, LWUE in WD1 and WD3 at seedling was significantly increased (*p*< 0.05) by 6.83% and 4.39% respectively compared to CK, while LWUE in WD2 had no significant difference (*p* > 0.05) compared to CK. In 2020, LWUE in WD1, WD2 and WD3 at seedling increased compared to CK, but the difference was not significant. For both 2019 and 2020, LWUE in severe water deficit treatments (WD5 and WD6) at seedling was significantly lower than CK. In addition, LWUE in all water deficit treatments at maturity was reduced compared to CK in 2019, but the reductions in WD2 and WD3 did not reach a significant level. In 2020, LWUE in mild water deficit treatments (WD1, WD3 and WD5) at maturity was not significantly different from CK, while that in the moderate water deficit treatments (WD2, WD4 and WD6) decreased significantly compared to mild water deficit treatments and CK.

### 3.2 Photosynthetic limiting factor

**Figure 6:**
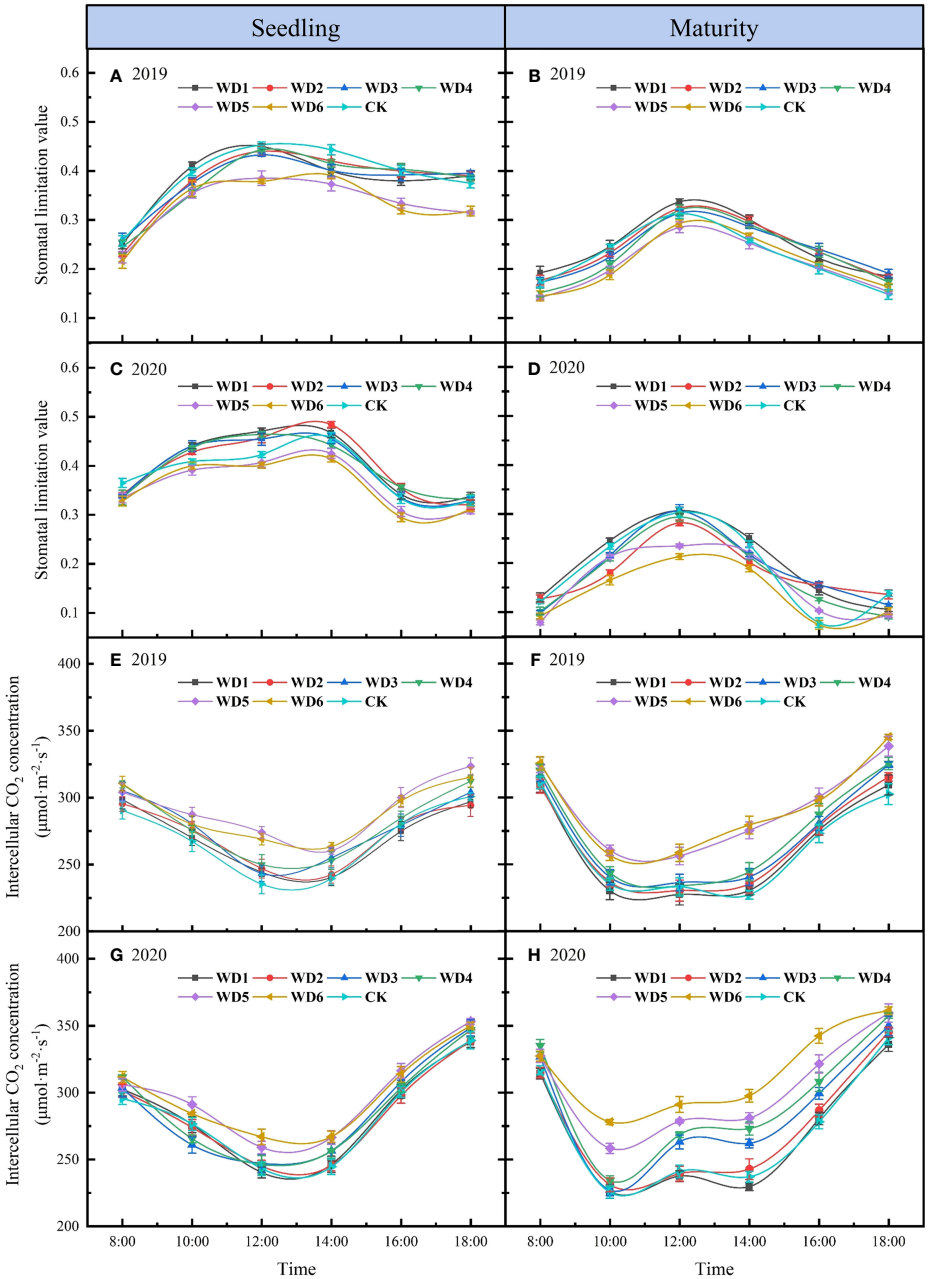
Daily variation of stomatal limitation value **(A–D)** and intercellular CO_2_ concentration **(E–H)** for different water deficit treatments in sunflower at seedling and maturity in 2019 and 2020. Bars indicate the standard deviations.

**Figure 6** shows the daily variation of Ls and Ci in sunflower during both seedling and maturity in 2019 and 2020. The daily variation in Ls demonstrated a general trend of initial increase followed by a decrease (**Figures 6A–D**), while Ci exhibited mainly a “V”-shaped trend, characterized by increased values in the morning and afternoon, and a reduction at midday. (**Figures 6E–H**). Analyzing the change direction of Ls and Ci, it is evident that Ci experienced a decline from 8:00 to 13:00 during seedling in 2019 and 2020, concomitantly with an upsurge in Ls, indicating that photosynthesis was mainly influenced by stomatal limitation during this period. Subsequent to 14:00, Ci gradually increased while Ls gradually decreased, indicating that the photosynthetic limiting factors gradually switched from stomatal to non-stomatal limitation post this period. At maturity, the duration wherein photosynthesis was influenced by stomatal limitation was shortened compared to that of the seedling, and it gradually switched from stomatal to non-stomatal limitation after about 12:00.

Observations from different deficit irrigation treatments reveal a decline in Ls at both seedling and maturity concomitant with increasing water deficit degrees in 2019 and 2020. Notably, Ls in WD5 and WD6 decreased significantly, especially during the seedling, with mean reductions of 11.26% and 15.00%, respectively, compared to CK in two years. Conversely, the Ci demonstrated an inverse variation to that of Ls, increasing with an intensified degree of water deficit. Ci values in WD1, WD2 and CK were basically the same and maintained at a lower level, while Ci in WD5 and WD6 were significantly increased compared to CK. These results suggested that stomatal limitation was the main factor affecting photosynthesis in sunflower leaves under mild water deficit conditions. However, as the degree of water deficit increased, particularly under severe water deficit conditions, non-stomatal limitation became the main factor affecting photosynthesis.

### 3.3 Yield components and irrigation water use efficiency

**Figure 7:**
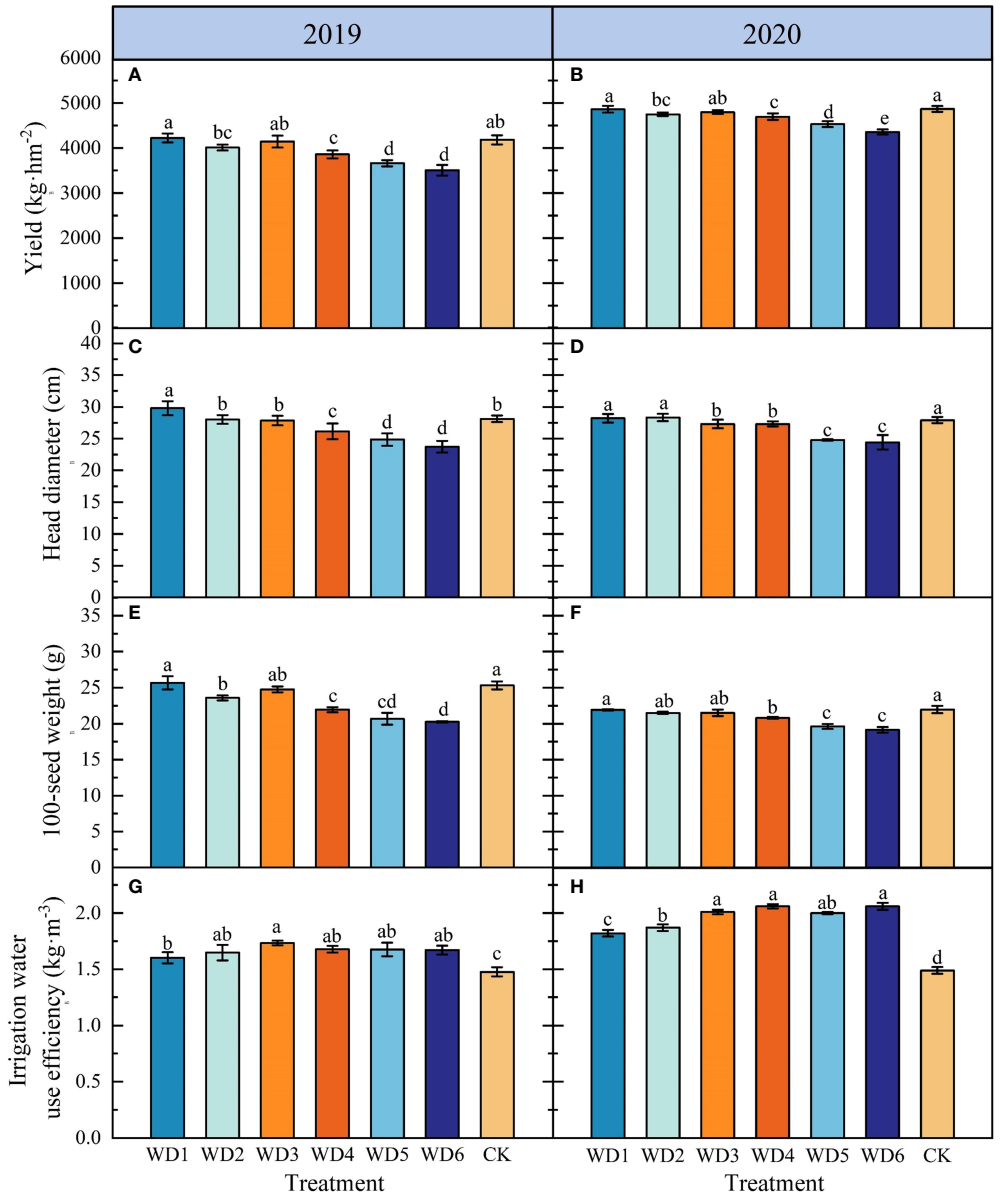
Effect of different water deficit treatments on sunflower yield **(A, B)**, HD **(C, D)**, HW **(E, F)** and IWUE **(G, H)** in 2019 and 2020. Different lowercase letters indicate significant differences at *p*< 0.05. Bars indicate the standard deviations.

The effects of different deficit irrigation treatments on sunflower yield, HW, HD and IWUE are shown in **Figure 7**. In 2019, yield, HW and HD of WD1 and WD3 were not significantly different (*p* > 0.05) compared to CK, while IWUE increased significantly (*p<* 0.05) by 8.46% and 17.33%, respectively. In 2020, while yield and HW of WD1 and WD3 were not significantly different compared to CK, IWUE increased by 22.15% and 34.90%, respectively. Furthermore, IWUE of WD2 was significantly higher by 11.49% and 25.50% compared to CK in 2019 and 2020, respectively, while the yield was lower by 4.07% and 2.43%, respectively. In addition, the two-year average increase of IWUE in WD4, WD5 and WD6 was 25.93%, 23.85% and 25.69%, respectively, compared to CK, but yield, HW and HD were significantly lower in these three treatments. These results indicated that water deficit irrigation was beneficial in increasing the IWUE of sunflower, but excessive water deficit could pose a risk of yield reduction. Notably, among all deficit irrigation treatments, WD1 and WD3 significantly increased IWUE and maintained yield.

### 3.4 Seed quality

**Figure 8:**
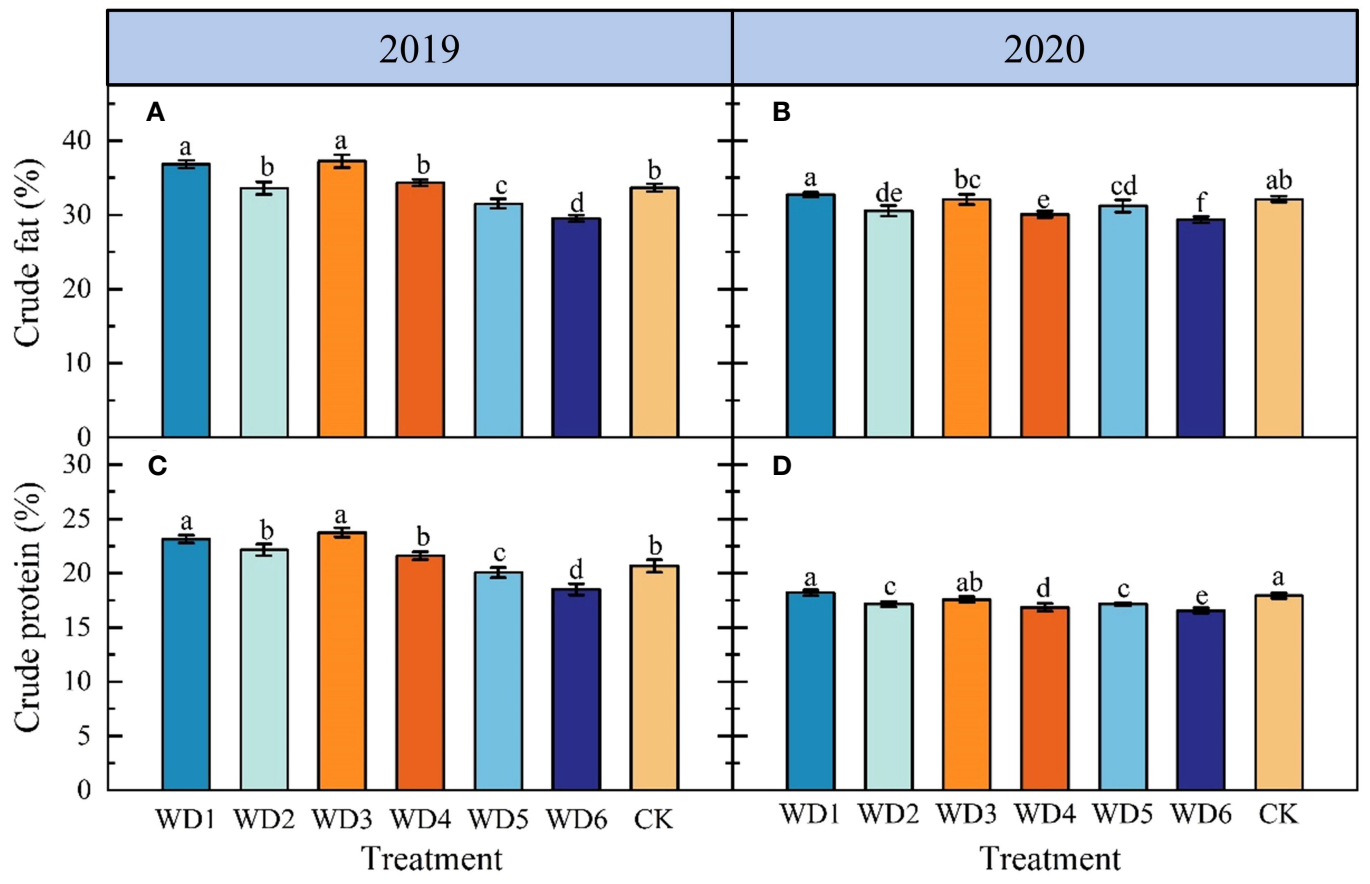
Effect of different deficit irrigation treatments on crude fat **(A, B)** and crude protein **(C, D)** content of sunflower in 2019 and 2020. Different lowercase letters indicate significant differences at *p*< 0.05. Bars indicate the standard deviations.

**Figure 8** illustrates the effect of each deficit irrigation treatment on sunflower seed quality. In 2019, there was a significant increase (*p*< 0.05) in CF and CP contents in WD1 by 8.22% and 12.13%, respectively, and in WD3 by 10.84% and 14.90%, respectively, compared to CK. In 2020, CF and CP contents of WD1 and WD3 were not significantly different (*p* > 0.05) from those of CK. Additionally, CF and CP contents in WD2 and WD4 did not show a significant difference to the CK in 2019, but were significantly reduced compared to CK in 2020. For both WD5 and WD6, CF and CP contents were significantly lower than those of CK over the two years, with WD6 showing the greatest reduction, averaging declines of 10.33% and 9.16%, respectively. These results suggest that a mild/moderate water deficit at seedling and a mild water deficit at maturity can achieve water saving and quality improvement to a certain degree, while a moderate water deficit at the maturity could reduce sunflower seed quality.

### 3.5 Comprehensive evaluation of sunflower deficit irrigation strategy

#### 3.5.1 Correlation analysis between evaluation indicators

**Figure 9:**
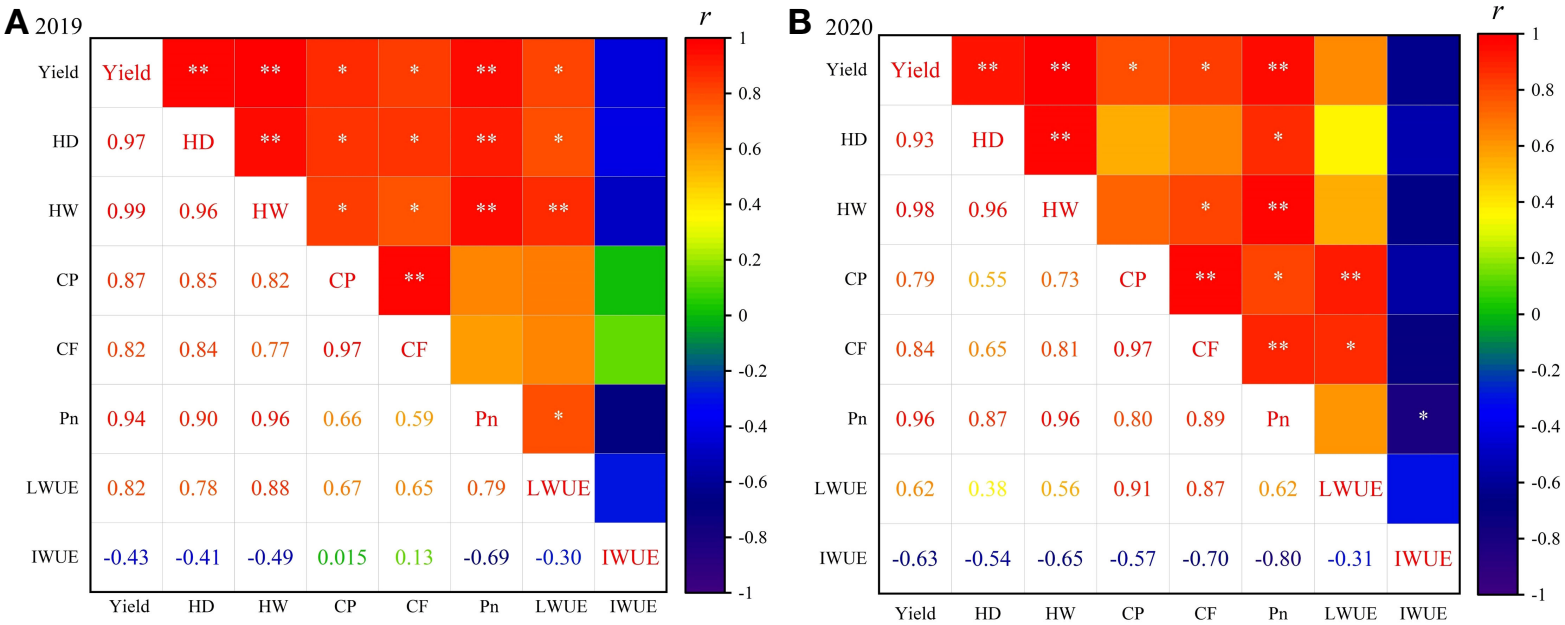
Correlation analysis between evaluation indicators in 2019 **(A)** and 2020 **(B)**. * and ** represent the significant differences at p< 0.05 and p< 0.01, respectively.

In the present study, eight variables including sunflower yield, HD and HW, CF, CP, Pn, LWUE and IWUE, were selected as evaluation indicators for the comprehensive evaluation of different water deficit treatments. Before establishing the evaluation system, a correlation analysis was conducted among all evaluative indicators (**Figure 9**). The two-year results showed that the yield was highly significantly positively correlated (*p*< 0.01) with HD, HW and Pn, with correlation coefficients greater than 0.9; yield also displayed a significant positive correlation with CP, CF and LWUE, and a weak negative correlation (*p* > 0.05) with IWUE; CF was significantly positively correlated with CP, HD and HW, whereas Pn was significantly negatively correlated with IWUE and positively correlated with other indicators. Additionally, various degrees of correlation were observed among other indicators. It can be seen that the information reflected by each indicator has some overlap and crossover due to the complex correlation between the indicators. Therefore, PCA can be used to transform highly correlated indicators into a new set of independent composite indicators, thereby providing a reliable and comprehensive assessment of the irrigation effectiveness of different water deficit treatments.

#### 3.5.2 Comprehensive evaluation

**Figure 10:**
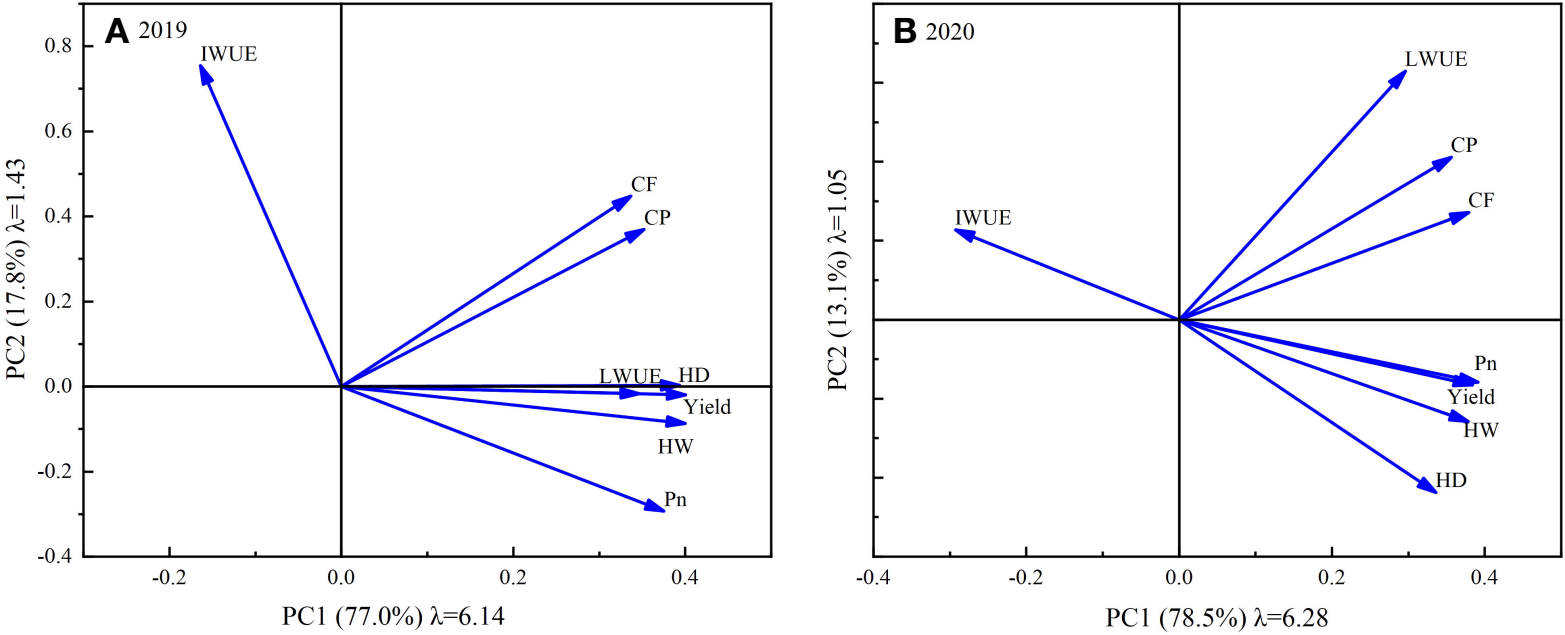
Principal component factor loadings, eigenvalues and variance contributions for 2019 **(A)** and 2020 **(B)**.

PCA was performed on the eight selected evaluation indicators. In both 2019 and 2020, two principal components were extracted according to the principle of eigenvalue greater than 1. The cumulative variance contribution rates of the two years were 91.64% (2019) and 94.61% (2020), respectively, indicating that the extracted principal components had high information representativeness. **Figure 10** shows the eigenvalues, variance contribution rates and factor loads of the principal components extracted in 2019 and 2020. As shown, the eigenvalues (λ) of the 1st principal component (PC1) were 6.14 and 6.28, and the variance contribution rates were 77.0% and 78.5% in 2019 and 2020, respectively. Conversely, the eigenvalues of the 2nd principal component (PC2) were 1.43 and 1.05, and the variance contribution rates were 17.8% and 13.1%, respectively.

Based on the score coefficients calculated from the eigenvalues and factor loads, the score values of the principal components extracted in 2019 and 2020 were calculated using Eq. (4) (**Table 2**). Considering the score values as mutually independent composite indicators, the membership function values [u(x)] for each treatment were calculated via Eq. (5). The weights of each composite indicator were subsequently determined using Eq. (6), whereby PC1 and PC2 were weighted at 0.86 and 0.14 in 2019 and 0.81 and 0.19 in 2020, respectively. Comprehensive score values (D) for each deficit irrigation treatment were subsequently determined utilizing Eq. (7). Treatments were ranked according to their respective D values, with a higher D value indicating a superior irrigation effect. As can be seen from **Table 2**, CK was ranked second and third, achieving D values of 0.693 and 0.888 in 2019 and 2020, respectively. Among all deficit irrigation treatments, WD1 secured the highest rank, with D values of 0.927 and 0.991 in 2019 and 2020, respectively, followed by WD3 with D values of 0.892 and 0.750, respectively, while WD6 had the lowest rank, with D values of 0.087 and 0.089, respectively. Therefore, WD1 is the best deficit irrigation strategy for sunflower, while WD3 can be used as a standby strategy in case of insufficient irrigation water.

**Table 2:** Principal component scores, membership function values and comprehensive scores of different water deficit treatments in 2019 and 2020.

Treatments	2019						2020					
	PC1	PC2	u(1)	u(2)	D	Ranking	PC1	PC2	u(1)	u(2)	D	Ranking
WD1	2.811	0.149	1.000	0.613	0.927	1	2.916	1.002	1.000	0.937	0.991	1
WD2	0.686	0.08	0.668	0.596	0.654	4	0.114	-1.555	0.591	0.000	0.507	4
WD3	1.958	1.668	0.867	1.000	0.892	2	1.131	0.657	0.739	0.811	0.750	3
WD4	-1.106	0.669	0.388	0.746	0.455	5	-1.132	-0.833	0.409	0.265	0.389	5
WD5	-2.64	0.155	0.148	0.615	0.236	6	-1.859	1.174	0.303	1.000	0.403	6
WD6	-3.588	-0.461	0.000	0.458	0.087	7	-3.936	0.151	0.000	0.625	0.089	7
CK	1.88	-2.26	0.855	0.000	0.693	3	2.766	-0.596	0.978	0.351	0.888	2

## 4 Discussion

### 4.1 Effects of water deficit on photosynthetic characteristics and its limiting factors

Investigating the response mechanisms of the photosynthetic attributes of plant leaves to water deficit can elucidate plant adaptations to water stress at the physiological level, thereby facilitating the development of more effective water management strategies. In this study, water deficit at both seedling and mature stages significantly reduced (*p*< 0.05) leaf Pn, Tr and Gs in sunflower, and the reductions increasing concomitant with escalating water deficit levels. Severe water deficit at seedling markedly suppressed leaf photosynthesis, precipitating a decrease in aboveground biomass and consequently maintaining leaf Pn, Tr and Gs at diminished levels even under mild water deficit at maturity. Many previous studies have reported that water deficit can inhibit photosynthesis in plant leaves, with the inhibitory effect becoming more pronounced as the degree of deficit increases. (Monneveux et al., 2006; Lawlor and Tezara, 2009; Chang et al., 2015). Yun et al. (2014) found that the photosynthetic capacity of sunflower leaves gradually weakened with decreasing soil water content, aligning with the findings of the current study. Additionally, the present study found that LWUE increased under the mild water deficit at both seedling and maturity compared with CK, while LWUE was significantly decreased under the severe water deficit at seedling and moderate water deficit at maturity. This indicated that under the mild water deficit treatment, the decrease of Tr was greater than that of Pn, which improved the LWUE by effectively suppressing Tr. However, with increasing water deficit, the decrease of Pn gradually became greater than that of Tr, resulting in a significant decrease in LWUE.

Stomatal limitation and non-stomatal limitation are the main factors through which water stress impacts photosynthesis in plant leaves. In the current study, it was observed that Ls at seedling and maturity showed an increasing trend around 8:00 to 13:00, and then gradually decreased, while Ci showed an opposite trend within the identical timeframe. This indicates that photosynthesis of sunflower leaves was mainly affected by stomatal limitation from morning to noon, transitioning gradually to non-stomatal limitation post-noon. The result was similar to the that of Liu et al. (2021a), who found that the decrease in leaf photosynthetic rate of alfalfa under water stress between 7:30 and 11:30 was attributed to stomatal limitation, which gradually changed to non-stomatal limitation post-11:30.

In this study, it was found that Ci in sunflower leaves increased with increasing degree of water deficit, while Ls changed in the opposite direction, indicating that with increasing degree of water deficit, the factors leading to the reduction of photosynthesis in sunflower leaves progressively transitioned from stomatal to non-stomatal limitation. Specifically, stomatal limitation is the main factor leading to a reduction in photosynthesis in sunflower leaves under mild water deficit conditions. Here, the stomatal closure of the leaves prevents external CO_2_ from entering the leaves, resulting in a CO_2_ concentration that does not meet the requirements of photosynthesis (Chaves et al., 2002). Conversely, under severe water deficit conditions, the decrease in photosynthesis of sunflower leaves was mainly due to non-stomatal limitation. In this scenario, excessive water stress can instigate the expansion and disarray of chloroplasts in leaves and damage the ultrastructure of photosynthetic organs (Liu et al., 2005). Moreover, a pronounced water deficit may also cause a reduction in photosynthetic pigment content and photosynthetic enzyme activities, coupled with a disruption of reactive oxygen metabolism functions, culminating in a reduction in photosynthesis. The results of this study are consistent with the findings of previous related studies. For instance, Zhang et al. (2018) showed that water deficit treatment could reduce Pn in citrus, and the main factors influencing Pn reduction gradually switched from stomatal to non-stomatal limitation as the increasing degree of water deficit. Campos et al. (2014) investigated stomatal and non-stomatal limitation of photosynthesis in sweet pepper leaves under water stress conditions, revealing that both stomatal closure and non-stomatal limitation together caused the decrease in photosynthesis under severe water stress.

### 4.2 Effects of water deficit on grain productivity and quality

In recent years, an increasing number of studies have focused on obtaining optimal economic productivity and resource use efficiency at lower cost to ensure sustainable agricultural development in arid areas (Kang et al., 2021; Li et al., 2022; Zhang et al., 2006). Many previous studies have shown that deficit irrigation can achieve the objectives of increasing crop yield and quality with less water input (Garcia-Tejero et al., 2010; Blanco et al., 2019; Gucci et al., 2019). The present study found no significant difference (*p* > 0.05) between the yields of WD1 and WD3 compared to those of CK; however, the IWUE of both was significantly higher (*p*< 0.05) than that of CK. Similar results were obtained by Karam et al. (2007), who found that water deficit at the early stage of seed formation could slightly increase sunflower yield and significantly improve WUE. Furthermore, both Connor et al. (1985) and Sadras et al. (1993) suggested that the increase in sunflower grain yield under water stress was due to an increase in the proportion of assimilates allocated to the head grain, alongside a decrease in translocation to the majority of vegetative organs. However, Kiani et al. (2016) revealed that both sunflower biomass and yield were consistently and significantly decreased with reduced irrigation water. This result differs from that of the present study, which may be due to the fact that the mulching used in this study has the potential to maintain soil moisture content and nutrient balance (Mubarak et al., 2021; Salem et al., 2021), thereby not significantly reducing yield (El-Metwally et al., 2022).

Previous studies have shown variations in nutrient uptake and distribution in plant parts under deficit irrigation conditions compared to normal irrigation (Saudy and El-Metwally, 2019; El-Metwally and Saudy, 2021). Consequently, significant changes occurred in the quality of economic products (Saudy and El-Metwally, 2023). In the present study, CF and CP contents increased in WD1 and WD3 treatments compared to CK, especially reaching significant levels (*p*< 0.05) in 2019, while CF and CP contents significantly decreased in the remaining water deficit treatments. Similarly, Ebrahimian et al. (2019) found that mild water stress treatment had no significant impact on the oil content of sunflower seeds, while severe water stress could significantly reduce the oil content. Petcu et al. (2001) showed that only the content of oleic acid in sunflower seeds was reduced under water stress conditions, while the content of other quality indicators did not change significantly. Therefore, applying an appropriate water deficit to sunflower will not reduce seed quality and may potentially enhance it.

### 4.3 Comprehensive evaluation of different water deficit treatments

The results of the two-year comprehensive evaluation based on PCA and MF methods showed that among all the deficit irrigation treatments, WD1 had the highest comprehensive score, followed by WD3, with WD6 achieving the lowest score. Thus, WD1 (mild water deficit at both seedling and maturity) can better achieve the triple objectives of stable yield, quality improvement and water conservation, and is the appropriate deficit irrigation strategy for sub-membrane drip irrigation of sunflower in the Hexi Oasis irrigation area of Northwest China. In scenarios where irrigation water is scarce, WD3 (moderate water deficit at seedling and mild water deficit at maturity) may serve as a backup strategy. Additionally, it should be acknowledgment that the comprehensive evaluation herein contains limitations, in that it does not account for the experiment’s costs and benefits, soil environment and certain other physiological and growth indicators of sunflower within the evaluation system, which may have some influence on the evaluation results. Therefore, the effects of deficit irrigation on costs and benefits, soil physicochemical properties and other indicators of stress resistance should be further investigated in the future to enhance the comprehensiveness and reliability of the evaluation system. Concurrently, given the substantial year-to-year and region-to-region variation in climatic conditions, future research should be conducted in multiple regions and years, so that deficit irrigation strategies suitable for efficient sunflower production can be developed according to local conditions.

## 5 Conclusion

Water deficit can exert inhibitory effects on leaf photosynthesis of sunflower, and these effects increased with the aggravation of water deficit. Mild water deficit at seedling and maturity improved LWUE and showed a more efficient water use strategy. Stomatal limitation was the main factor leading to the decrease in photosynthetic capacity of sunflower leaves under mild water deficit, whereas non-stomatal limitation became the dominant factor under severe water deficit. In addition, in comparison to full irrigation, mild/moderate water deficit at seedling and mild water deficit at maturity (WD1 and WD3) increased CF and CP contents and maintained yield, while IWUE increased significantly (*p*< 0.05). Comprehensive evaluations employing PCA and MF methods indicated that a mild water deficit at both seedling and maturity (WD1) could well achieve the efficient combination of stable yield, superior quality and water saving, thereby emerging as the optimal deficit irrigation strategy for sunflower at the study area. Consideration could be given to a moderate deficit at seedling and mild deficit at maturity (WD3) as an alternative strategy when irrigation water is insufficient. Nonetheless, it should be noted that there are differences in climate and soil conditions in different arid regions, and the appropriate deficit irrigation treatment in this study may not be the optimal water management strategy for other arid regions. Therefore, future multi-regional studies should be conducted to investigate the response of sunflower water productivity, seed quality and economic efficiency to water deficit under varying climatic and soil conditions. According to local conditions, a deficit irrigation strategy is proposed to help farmers reduce irrigation inputs and ensure sustainable and efficient agricultural production in arid areas.

## Data availability statement

The original contributions presented in the study are included in the article/supplementary material. Further inquiries can be directed to the corresponding author.

## Author contributions

XC: Data curation, Formal Analysis, Writing – original draft, Writing – review & editing. HZ: Funding acquisition, Supervision, Writing – review & editing. AT: Formal Analysis, Writing – review & editing. CZ: Data curation, Writing – review & editing. LL: Data curation, Writing – review & editing. YB: Formal Analysis, Writing – review & editing. ZW: Formal Analysis, Writing – review & editing.
